# Knowledge, attitudes and professional practices of ortho-periodontal care of adults: a cross-sectional survey in France

**DOI:** 10.1186/s12903-022-02177-3

**Published:** 2022-04-26

**Authors:** Apolline Saloux, Antoine Couatarmanach, Brice Chauvel, Sylvie Jeanne, Damien Brezulier

**Affiliations:** 1 CHU Rennes, Pole Odontologie, Univ Rennes, 2 Av. du Professeur Léon Bernard, Bât.15, 35043 Rennes Cedex, France; 2 Arènes, CNRS – UMR 6051, 35000 Rennes Cedex, France; 3 LTSI, CNRS – UMR 1099, 35000 Rennes Cedex, France; 4 ISCR, CNRS – UMR 6226, 35000 Rennes Cedex, France

**Keywords:** Adult orthodontics, Periodontics, Survey, Professional practices

## Abstract

**Background:**

Due to increasing numbers of adult patients, orthodontists are being confronted more and more with periodontal problems. Coordination amongst orthodontists, periodontists and general dentists is useful in preventing and stopping periodontal disease. The main objectives of this survey were to evaluate the technical knowledge, techniques and attitudes employed by French orthodontists, periodontists and general dentists in adult dental care.

**Methods:**

A cross-sectional online survey was distributed to French dentists. The questionnaire, consisting of 30 questions, was divided into six sections covering treatment programs and the forensic environment.

**Results:**

One thousand one hundred twenty-two complete answers were recorded. Adults undergoing orthodontic treatment represented 19.9% of the orthodontists' patients, but only 2.67% of the general dentists' patients. Communication between clinicians was rated as good, greater than 3 out of 5. Before treatment, orthodontists were less alarmed than generalists regarding bleeding, recessions, increased probing depths, halitosis and hyperplasia. During treatment, orthodontists never or only occasionally performed palpation or probing in 54.2% and 84.6% of cases. Gingivitis and recessions were the main reasons for consultations for 22.0% and 20.1% of general dentists and periodontists after orthodontic treatment. Of the practitioners surveyed, 43% felt that they experienced a setback in the ortho-periodontal treatment.

**Conclusions:**

This study revealed discrepancies in the knowledge and attitudes of practitioners. Therapeutic management remains one of the major challenges of multidisciplinary treatments. Continuing education needs to be further developed in this field.

## Introduction

Recent years have seen a sharp increase in adult orthodontic treatment rates. According to the American Association of Orthodontics (AAO), the average number of treated adults in the U.S. increased by 7% between 2016 and 2018, an all-time high. Today, one in three patients are over the age of 18 [1]. Patients have an increasing desire to align their teeth in order to improve their smile due to societal pressures and the relentless quest for normalcy [2].

To meet their expectations, it is necessary to consider the aging of the teeth: worn, restored or missing teeth and periodontal problems. Current WHO data show an average DMFT index of more than 13.9 for adults aged 35–44 years in France [3]. The number of patients in this age group with one or more indirect restorations is estimated at 9.1% [4]. The U.S. National Health and Nutrition Examination Survey (NHANES) completed in 2010 reveals that a 47% prevalence of periodontitis is observed in adults over 30 years of age following the emergence of dysbiosis in a permissive host [5]. Age-related immune and cellular senescence and impaired healing directly affect the severity of periodontal disease [6–8]. Thus, 80% of adults suffer from gingivitis or mild to moderate periodontitis (stage I or II) and it is even suggested that one-tenth suffer from severe periodontitis (stage III or IV) [9]. Periodontitis is the sixth most common chronic disease [10]. In summary, the number of patients with periodontal disease that are treated with orthodontics is higher [11, 12].

Periodontology and orthodontics are intimately connected. Crowding and occlusal trauma can jeopardize the periodontium. The expected results of orthodontic treatment are the maintenance of good oral hygiene, the elimination of occlusal trauma, a better distribution of inter-root spaces and the improvement of the prognosis of mucogingival surgery [8, 12–15]. However, it is important to be wary of the iatrogenic risk of orthodontics on the periodontium. Fixed orthodontic appliances, by quantitatively and qualitatively modifying the oral microbiota, increase the proportion of anaerobic bacteria and promote gingival inflammation [16]. On a healthy periodontium, even if weakened, orthodontic forces maintained within biological limits (respect for bone anatomy, pressure on soft tissues) do not cause gingival inflammation if oral hygiene remains effective. Unfortunately, these forces aggravate the disease in patients with periodontitis, even with good oral hygiene [17]. Periodontal disease is only a temporary contraindication to orthodontic treatment. It disappears when periodontal disease is treated. The systemic review carried out by the 2014 AAP (American Association of Periodontology) Regeneration World Workshop reports that the direction of tooth movement and gingival thickness play an important role in soft tissue alterations during orthodontic treatment [18]. Some movements, such as a buccal tipping or excessive expansion, lead to thinning of the buccal cortical bone and even bone dehiscence in a healthy periodontium. In contrast, soft tissue response is less predictable and multiple factors influence it [19]. The literature remains conflicting on the role of orthodontic treatment in the development of gingival recession. The authors report an increase in long-term prevalence of up to 47% [20–23]. Several risk factors have been identified: bone anatomy marked by thin cortical surfaces or even fenestrations or dehiscence, thin biotype, poor oral hygiene, and inappropriate orthodontic mechanics [13].

In this context, prevention of periodontal and dental risks associated with orthodontic therapy in adults becomes paramount. Two stages can be distinguished [24]. The first is the establishment of oral hygiene techniques adapted to orthodontics. The second is based on professional maintenance. It is based on a rigorous follow-up of the periodontal parameters by probing, palpation or retro-alveolar X-ray. Nevertheless, these examinations are the key to the detection of periodontal anomalies [25, 26]. Bleeding on probing is the most reliable diagnostic method for assessing gingival inflammation [27, 28]. The study of radiographs along with probing provides very sensitive detection of attachment and bone loss [9]. Moreover, palpation, from the bottom of the vestibule to the occlusal direction, is the best examination to detect any suppuration, a sign of periodontal disease activity and tissue destruction [29].

In the clinic, a contribution between the orthodontist, the periodontist and the general dentist is essential to optimize treatment results. If all are able to detect periodontal anomalies, only general dentists and periodontists can repair them. Currently, exacerbation of periodontal problems during orthodontic treatment is a major cause of malpractice litigation in the United States [30]. Practitioner responsibility is shared for the design of the treatment plan, but it remains individual for each practitioner in the performance of his or her specific part of the treatment.

The coordination between clinicians allows the interception or prevention of disease, the integration of the patient into a maintenance program and the possible indication of a periodontal reinforcement according to the scheduled tooth movements. However, this ideal care is based on the hypothesis that the different protagonists have an equivalent level of knowledge on this subject. In this context, the objective of this cross-sectional survey was to evaluate the knowledge, attitudes and professional practices of French orthodontists, general dentists and periodontists during the ortho-periodontal management of adults.

## Materials and methods

### Dissemination

The survey was disseminated to French dentists and orthodontists in four ways. Each one provides a URL link: (i) a manual search in the directories of scientific societies; (ii) e-mail campaign by the County Councils of the Order, professional unions, Regional Union of Health Professionals; (iii) sending paper mail containing QR-codes to dental laboratories that agreed to redistribute them to their clients; (iv) distribution on the social network Facebook via professional pages.

The privacy policy of the survey ensured that all data collected were stored and processed anonymously on the LimeSurvey server. The platform's settings have been made compliant with the standards of the General Data Protection Regulation, i.e. no IP recording and no time stamping of participation. The approval of an ethics committee was not necessary given the type of study. The declaration has therefore only been made to the data protection officer of the University of Rennes 1. Data collection occurred from September 17, 2020 to January 7, 2021.

### Survey

#### Construction

Searches on PubMed and Google scholar were conducted with the key words: "adult", "orthodontics", "periodontal disease", and "complications". It led to the design of a self-administered double-entry questionnaire made available via the online survey platform LimeSurvey by the author (AS). The first entry was for orthodontists and practitioners with a predominantly orthodontic practice, hereafter "orthodontists". The second entry was for general dentists and periodontists exclusively, referred to as "general dentists" and "periodontists" respectively. The validity of the questionnaire was tested by a pilot group asked to review both the content and format and assess the relevance to their prevailing activity. Suggestions were compiled for improvement.

#### Structure

The questionnaire was divided into six sections covering the different times of treatment and the medico-legal context: (i) description of the practitioner sample, (ii) of the patient base, (iii) evaluation of the management before, (iv) during, (v) after orthodontic treatment, (vi) study of failures of ortho-periodontal treatment. It included 30 questions, 18 of which were addressed to general dentists and periodontists and 19 to orthodontists.

#### Method

Three interrogative modalities coexisted in the questionnaire. The first form is the "point and click" one, known for its simplicity. The answers were arranged in the form of "radio buttons" allowing a single choice to avoid inconsistencies. These were either closed questions (yes/no) or Likert scales in interrogative form, with an even number of verbatim responses to eliminate any centrist position. With the exception of the scale assessing communication, which was numerical to be treated as a quantitative variable. The second form was based on numerical entry type questions. The third form consisted of single or grouped multiple choice questions. In the latter case, the question behaved like a table with several simple multiple-choice sub-questions. To reduce inconsistencies, some questions were conditioned. For example, if the answer to *"Do you perform periodontal probing?"* was negative, it was not proposed to diagnose an increased periodontal probing depth in the next question.

### Statistical analysis

Data obtained from the Lime Survey platform were exported into a Microsoft Excel® spreadsheet and analyzed for the three groups. Statistical analysis was performed with RStudio® software version 1.4.1103 (RStudioTeam) in R language version R 4.0.2 (RCore Team). Qualitative data were analyzed by Pearson's χ^2^ test with Yates' continuity correction. The Shapiro–Wilk test and Q-Q plot reading were used to determine the normality of the distribution of quantitative data. Group means were compared by Student t-test or ANOVA. Internal validity of Likert scale questions was checked by calculation of Cronbach's alpha. *P* values ≤ 0.05 were considered statistically significant.

## Results

### Description of respondents and their patient base

The distribution of the questionnaire was proposed to all the French County Councils of the Order. Of these, 45 responded positively, 21 negatively and 34 did not respond. The questionnaire was also proposed to 57 dental laboratories. Between September 17, 2020 and January 7, 2021, 1757 responses were recorded. 635 were only partial and were excluded. The sample consisted of 1122 complete responses. The participant panel included 622 women and 500 men with respective mean ages of 41.6 ± 11.4 years and 46.9 ± 12.8 years (*p* < 0.0001). The distribution of the prevailing activity was 536 orthodontists, 542 general dentists, and 44 periodontists. Adults undergoing orthodontic treatment, represented 19.9% of orthodontists' patients, but only 2.67% for general dentists and 11.4% for periodontists (*p* < 0.0001).

### Attitudes and practices

#### Before orthodontic treatment

First, the frequencies and modalities patient referrals were studied. Orthodontists were consulted on whether to refer patients to general dentists or periodontists based on age. On average, 86.9% of orthodontists referred their patients for a dental consultation. Patients under 30 years of age were mainly referred to the general dentist. Between the ages of 30 and 50, half of the patients were referred to the periodontist. In contrast, after 50 years of age, more than 72.2% were referred to the periodontist. However, only 7.91% of general dentists and periodontists referred their patients to the orthodontist after periodontal treatment. Most practitioners would refer to a colleague they used to work with. It was the case for 58.0% of orthodontists and 59.0% of general dentists and periodontists. The referral involves the transmission of medical information. The overall quality of referral was rated from 1 (none) to 5 (excellent). Orthodontists rated it as good with a score of 3.55 ± 0.92. The periodontists rated the quality of the discussions with the orthodontists as 3.64 ± 0.89. However, general dentists were more pessimistic, rating it at 3.35 ± 1.13 (*p* < 0.01). The orthodontic treatment plan should be discussed between clinicians. General dentists and periodontists were informed in 77.1% of cases. However, only 66.2% of orthodontists were informed about periodontal stability before orthodontics (*p* < 0.0001). The methods of exchange varied according to the prevailing activity (*p* < 0.0001): general dentists and periodontists were informed in 93.4% of cases by mail or e-mail, and in 5.3% by the patient only. Orthodontists were informed of the stabilization of periodontal disease by mail or e-mail in 71.0% of cases, and by the patient himself in 26.2%.

**Fig. 1:**
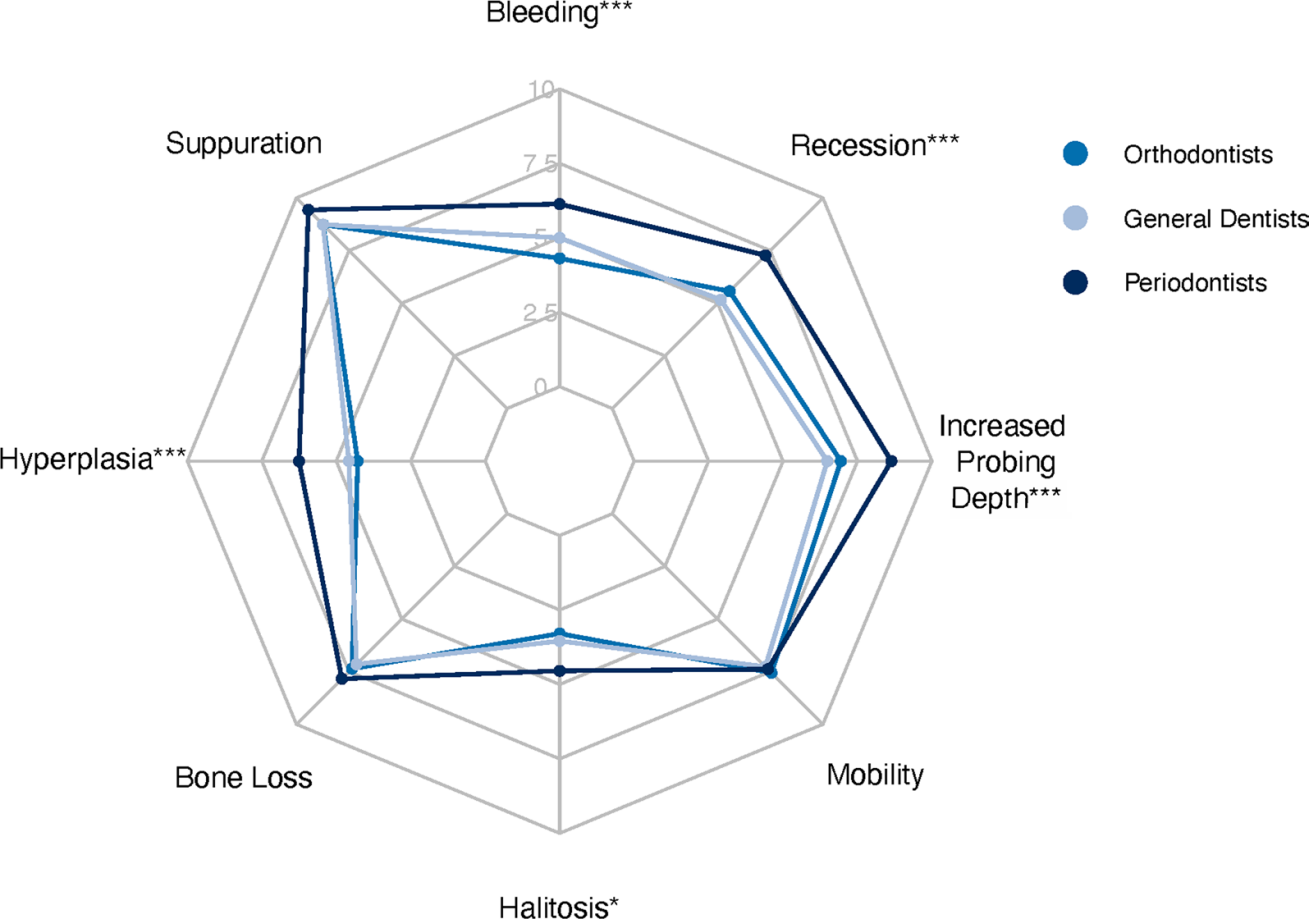
Kiviat diagram representation of degrees of treatment necessity for eight symptomatologies after transformation of qualitative variables into quantitative variables according to the following rule: "not necessary" equals 0, "relative degree of need for treatment" scores 5, "high degree of need for treatment" scores 10. For each symptomatology, the frequencies were compared by Pearson's χ^2^ test with Yates continuity correction. ***: *p* < 0.0001; *: *p* < 0.05

Second, professional attitudes regarding the degree of necessity of management of eight periodontal symptomatologies were assessed. For each group, the internal consistency of the response scales was good with a Cronbach's alpha above 0.70. The response patterns were similar for the suppuration, bone loss and mobility items. It was not the case for bleeding, recessions, increased probing depths, halitosis and hyperplasia, showing significant differences. For all these cases, periodontists were the most alarmist, while orthodontists and general dentists judged the need for treatment to be comparable (Fig. 1).

Third, information about the periodontal risks of orthodontic treatment was studied. Responses to the question *"Who has the duty to inform about the risks involved?"* varied. Orthodontists felt that 34.1% of the duty was theirs alone, while 60.1% partnered with general dentists or periodontists or both. General dentists felt that this duty was shared by all (52.2%). Finally, periodontists felt that the transmission of information was never the exclusive responsibility of orthodontists. In 79.5% of cases, the periodontists were involved in the transmission of information with or without the general dentists. Information modalities were also evaluated. All periodontists, and more than 99% of orthodontists, informed their patients. In contrast, 9% of general dentists did not provide information (*p* < 0.0001). For general dentists (63.6%) and for periodontists (69.6%) the information was exclusively oral (*p* < 0.0001). Only 29.7% of orthodontists used this modality. They used standardized written information alone (10.3%) or combined with oral information (27.6%). Some combined personalized written information with oral information (9.9%).

#### During orthodontic treatment

**Fig. 2:**
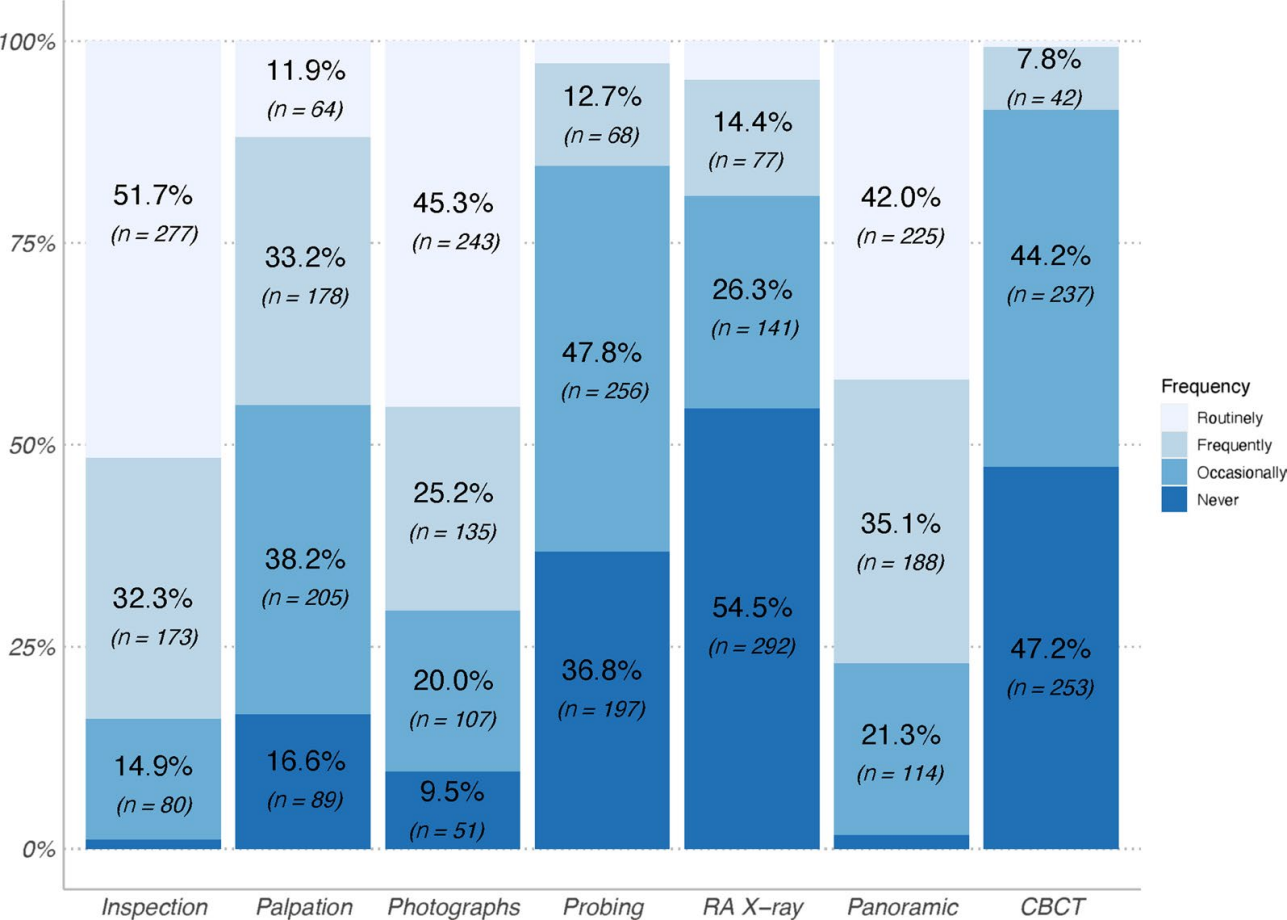
Frequency of use of different screening methods during orthodontic treatment by orthodontists. RA X-ray: retro-alveolar radiography, CBCT: cone beam computed tomography

Orthodontists' frequency of use of screening tools during treatment was variable (*p* < 0.0001). Inspection, photographs and orthopantomogram were performed by 84.0%, 70.5%, and 77.1% of orthodontists, respectively, routinely or frequently. In contrast, non-invasive examinations, such as palpation and probing were not performed, or were performed only occasionally by 54.2% and 84.6% of them. Other radiographs, such as retro-alveolar and 3D examinations, were regularly indicated by only 19.3% and 8.5% of orthodontists. Note that 54.5% of orthodontists never performed retro-alveolar X-ray in adult orthodontics (Fig. 2).

During orthodontics, bleeding, increased tooth mobility and hyperplasia were the most common symptoms detected by orthodontists. They were detected by 39.8%, 27.4%, and 36.8% of them, respectively, always or frequently. Halitosis, bone loss, recession and increased probing depths were encountered only occasionally or never by 93.9%, 97.6%, 95.2% and 96.1% of orthodontists. Suppuration was the least encountered, and absent for 69.2%.

Periodontal maintenance habits of periodontists and general dentists differed with active orthodontic treatment (*p* < 0.0001). Although follow-up every 6 months was the majority, the frequency was increased in the case of active appliances.

#### At the end of the treatment

44.1% of orthodontists referred for periodontal reassessment after orthodontic treatment. For general dentists and periodontists, tooth loss, exacerbated mobility and periodontitis were only a small proportion of complications. These were encountered by 1.5%, 6.2%, and 6.7% of general dentists and periodontists, respectively, on a systematic or frequent basis. In contrast, gingivitis and recessions were major reasons for consultation for 22.0% and 20.1% of them.

### Ortho-periodontal treatment failures

When asked the closed-ended question, *"Have you ever had an interdisciplinary ortho-periodontal treatment failure?"*, 43% of practitioners felt they had encountered such a situation. The distribution varied by prevailing activity (*p* < 0.0001).

**Fig. 3:**
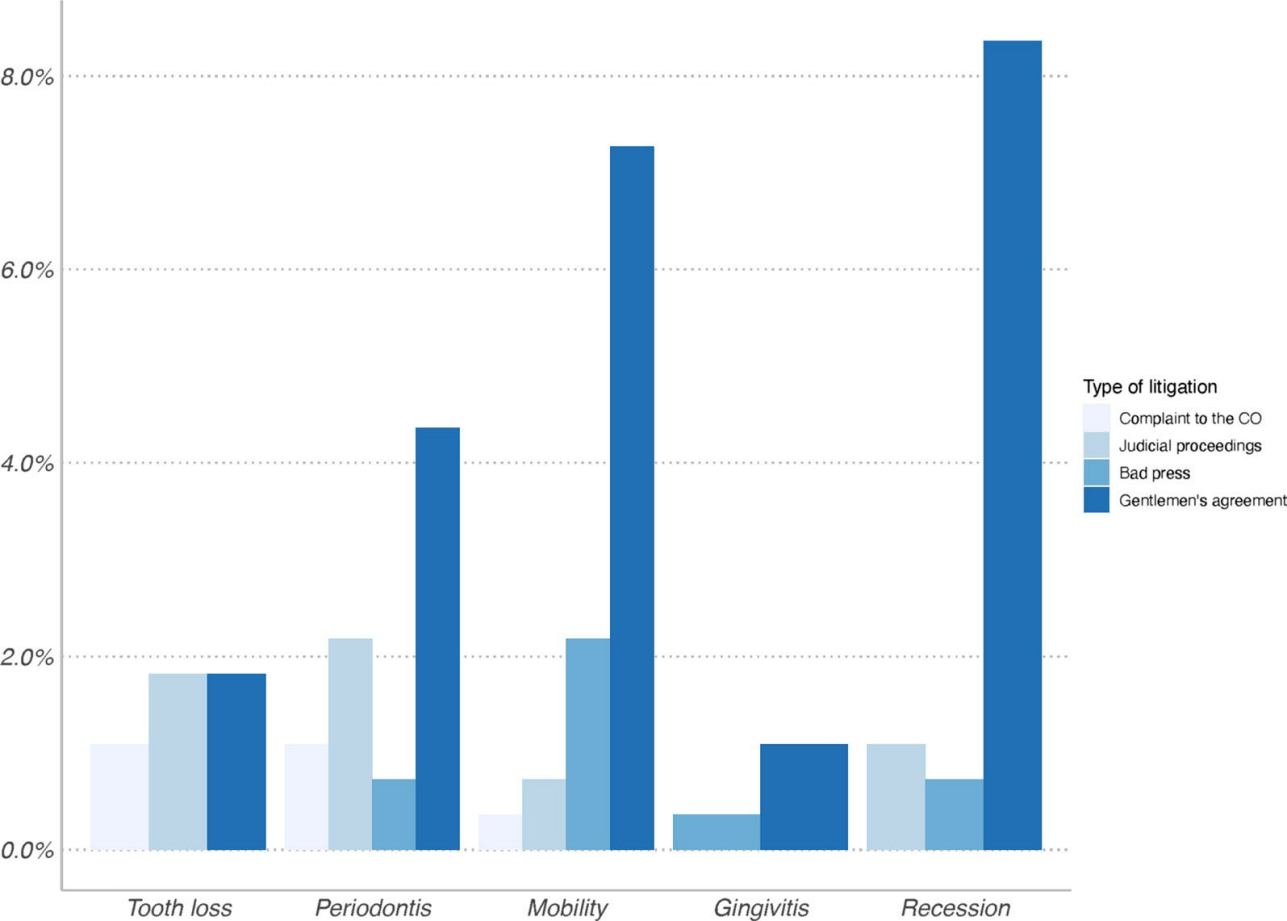
Percentage of orthodontists who have experienced litigation and type of litigation. OC: Council of the Order

Specifically, most orthodontists and periodontists had experienced this at least once. In contrast, only 33.6% of general dentists had experienced it. For orthodontists, the symptomatologies leading to litigation were increased mobility, periodontitis and recession. Among the amicable or judicial recourses, it is important to note the significant percentage of bad publicity made on the internet and on social networks. This bad press was found for all grievances, except for dental losses (Fig. 3).

The conflicts involved the patient/practitioner relationship and not the relationship between practitioners. These disagreements were the consequence of no-shows at appointments for 65.4%, poor compliance for 87.3%, lack of supervision during treatment for 60.4% and lack of information for 56.8% of all the clinicians. Poor communication, divergence about periodontal status or extractions were sources of litigation for only 46.1%, 34.9%, and 32.6% of them.

## Discussion

The construction of this questionnaire, its structure and the survey methods used were chosen for their proximity to other studies in dentistry [31–33]. The distribution of the questionnaire resulted in a high response rate, comparable to other studies conducted in Europe [34–36]. The 1122 participants represented 2.6% of French dentists. Of these, more than 17% of dentofacial orthopedics specialists responded, further strengthening the validity of the sample.

The frequency of orthodontic treatment of adults was extremely variable. On average, one in five orthodontic patients treated was an adult. Simultaneously, they represented only a small percentage for general dentists and periodontists. This raises questions about the knowledge and practices related to the follow-up of adult patients with orthodontic appliances. Moreover, less than 8% of general dentists and periodontists refer to the orthodontist after periodontal treatment. This finding shows the lack of knowledge among French dentists about the possibilities of periodontal improvement offered by orthodontics. The rest of the survey focused on knowledge and practices at key stages of patient care.

At first, these were evaluated before the start of orthodontic treatment. It is at this time that the screening for periodontal problems should occur, especially since the incidence of periodontitis peaks around the age of 38 [37]. In this context, it should be noted that most orthodontists referred adults for pre-screening. The link between age and periodontal disease is known to orthodontists. It is a decisive factor for referral to a general dentist or a periodontist with a cut-off point of 50 years.

The survey also showed good communication between orthodontists and general dentists and periodontists. Most orthodontists refer their adult patients to a particular colleague. This choice to limit the referral to a trusted practitioner was also found for general dentists and periodontists when orthodontic advice was needed. This approach ensured overall patient satisfaction. Indeed, it is past successful collaborative experiences that make practitioners prefer to refer their patients [38, 39]. Moreover, this type of orientation ensures an overall quality of communication judged as "good" by all protagonists (above 3 out of 5).

The high percentage of orthodontists informed of the periodontal status, or of general dentists and periodontists informed of the orthodontic treatment plan, testifies to the good quality of the exchanges. However, the method of communication differed according to the prevailing activity. Orthodontists mostly delivered the information in writing, whereas periodontists and general dentists communicated by mail, or through the patient himself in more than one case out of four. It is consistent with the literature, which shows that mail is the preferred method of communication between colleagues [31, 40].

Despite good communication, practitioners did not assign the same degree of need to treat to symptomatologies characteristic of periodontal problems [41]. Orthodontists are less alarmist than general dentists or periodontists about bleeding, gingival recession, hyperplasia, increased probing depths and halitosis. Moreover, during the treatment, the orthodontists did not perform an ideal follow-up: no probing, palpation or retro-alveolar X-ray. There are two possibilities: either they refer to the general dentist or the periodontist, or they do not seek information about it. Nevertheless, these examinations are the key to the detection of periodontal anomalies [25, 26].

This lack of rigorous follow-up or lack of feedback from practitioners, as only 66% of orthodontists were informed about periodontal stability, explains the frequent appearance of periodontal symptoms during orthodontic treatment. It is the case of gingivitis and increased mobility. Mechanical or chemical irritation caused by fixed appliances is responsible for inflammation. The biofilm initially supra-gingival becomes trapped in the sub-gingival area. The patient cannot clean these areas caused by the hypertrophy without the assistance of the orthodontist [42]. By contrast, periodontists reinforce the follow-up of patients undergoing orthodontics. The frequency of follow-up is increased.

This lack of vigilance results in an increased frequency of gingivitis and recessions after orthodontic treatment, even though they were already detectable during orthodontic treatment. Several studies report the observation of gingival recession after orthodontic treatment (mainly due to tipping of the mandibular incisors or maxillary expansion). Noted that the results are sometimes contradictory, with the reported prevalence ranging from 5 to 12% at the end of treatment [23, 43, 44]. Long-term prevalence reaches 47% [20–22]. It can be explained by the presence of predisposing factors: thinness of the cortical bone and its proximity to the roots, thin periodontal phenotype, unfavorable functional matrix, or poorly controlled environment. This is the case of deficient oral hygiene, bad habits, poor orthodontic mechanics exceeding the envelope of movements defined by Epker, … [13].

Although communication within the medical team is reported as good, orthodontists seem to be less vigilant about the evolution of periodontal conditions. This results in both symptomatology and litigation. For example, in the United States, the most common malpractice claims against orthodontists are related to the onset or development of periodontal problems [45]. The results of this survey show that more than half of the orthodontists and periodontists have already experienced the failure of multidisciplinary treatment. The vast majority reported that they had reached an amicable solution with the patient. Of note is the growing prominence of bad publicity for practitioners. The number of online reviews of doctors is increasing rapidly. One study found that in the USA, 80.9% of orthodontic practices have Google reviews [46]. This is higher than the 74% of businesses across all industries. Patient dissatisfaction is expressed through the notions of quality of care, interpersonal relationships as well as financial considerations [46, 47]. In addition to litigation for periodontal damage, missed appointments, lack of patient compliance, poor treatment monitoring or lack of information have led more than half of the clinicians to fail. This last point is crucial since obtaining informed consent appropriate to each patient is a prerequisite for any treatment [48]. However, this study found that 9% of general dentists do not inform about the periodontal risks of orthodontic treatment. In addition, whatever the prevailing activity, the information is only given orally.

In an increasingly litigious society, it is prudent to implement simple risk management strategies with the dual purpose of improving treatment quality and minimizing exposure to potential lawsuits. In this regard, better screening for risk situations should be performed by orthodontists as recommended by the AAO and the AAP [30]. This requires two things. The first is the use of adequate equipment such as graduated periodontal probes and generators for intraoral radiography. The second is the need to improve training. It seems appropriate to strengthen the continuing education of professionals on this topic. The results of this study illustrate the need to adapt education specifically to the prevailing activity. Knowledge and vigilance will then be the guarantors of the principle: *primum non nocere*.

## Conclusion

This cross-sectional survey of French dentists and orthodontists revealed disparities in the orthodontic and periodontal management of adult patients. Thus, three key concepts were highlighted:

- General dentists, periodontists and orthodontists do not assign the same degree of need to treat to clinical situations encountered before orthodontic treatment. Periodontists were the most alarming.
- Orthodontists perform limited periodontal screening. However, dentists frequently see patients for periodontal damage following orthodontic treatment.
- More than half of all practitioners have experienced ortho-periodontal treatment failure.

In addition, this survey highlights the need to strengthen academic and continuing education in this field. This should be done with general dentists as well as orthodontists and periodontists and should cover basic and applied concepts.

## Data Availability

The data underlying this article will be shared on reasonable request to the corresponding author.
